# Investigating the added value of incorporatingmammographic density to an integrated breastcancer risk model with questionnaire-based riskfactors and polygenic risk score

**DOI:** 10.21203/rs.3.rs-5445786/v1

**Published:** 2024-12-19

**Authors:** Charlotta V. Mulder, Xin Yang, Yon Ho Jee, Christopher G. Scott, Chi Gao, Yu Cao, Amber N. Hurson, Mikael Eriksson, Celine M. Vachon, Per Hall, Antonis C. Antoniou, Peter Kraft, Gretchen L. Gierach, Montserrat Garcia-Closas, Parichoy Pal Choudhury

**Affiliations:** National Cancer Institute; University of Cambridge; Harvard TH Chan School of Public Health; Mayo Clinic; Harvard TH Chan School of Public Health; American Cancer Society; National Cancer Institute; Karolinska Institutet; Mayo Clinic; Karolinska Institutet; University of Cambridge; National Cancer Institute; National Cancer Institute; Institute of Cancer Research; National Cancer Institute

**Keywords:** Breast Cancer, Mammographic breast density, Risk prediction model validation

## Abstract

**Introduction:**

Incorporation of mammographic density to breast cancer risk models could improve risk stratification to tailor screening and prevention strategies according to risk. Robust evaluation of the value of adding mammographic density to models with comprehensive information on questionnaire-based risk factors and polygenic risk score is needed to determine its effectiveness in improving risk stratification of such models.

**Methods:**

We used the Individualized Coherent Absolute Risk Estimator (iCARE) tool for risk model building and validation to incorporate density to a previously validated literature-based model with questionnaire-based risk factors and a 313-variant polygenic risk score (PRS). The model was evaluated for calibration and discrimination in three prospective cohorts of European-ancestry women (1,468 cases, 19,104 controls): US-based Nurses’ Health Study (NHS I and II) and Mayo Mammography Health Study (MMHS); and Sweden-based Karolinska Mammography Project for Risk Prediction of Breast Cancer (KARMA) study. Analyses were done separately for women younger (NHS II, KARMA) and older than 50 years (NHS I, MMHS, KARMA). Improvements in terms of risk stratification and reclassification proportions were assessed among European-ancestry women aged 50–70 years in US and Sweden.

**Results:**

For women younger and older than 50 years, the model with questionnaire-based risk factors, PRS and density was generally well calibrated across risk with some evidence of miscalibration at the extremes of the risk distribution. Incorporation of density led to modest improvements risk discrimination beyond the model with questionnaire-based risk factors and PRS: the area under the curve (AUC) among younger women was 67.0% (95% CI: 63.5–70.6%) vs. 65.6% (95% CI: 61.9–69.3%) for models with and without density; and 66.1% (95% CI 64.4–67.8%) vs. 65.5% (95% CI: 63.8–67.2%) among older women. The model with density identified 18.4% of US women 50–70 years old ≥ 3% 5-year predicted risk (threshold used for recommending risk-reducing medication in the US), with 42.4% of future cases expected to occur in this group. At this threshold, 7.9% of US women were reclassified by adding density to the model, resulting in the identification of 2.8% of additional future cases. The model with density identified 10.3% of Swedish women ≥ 3% 5-year predicted risk, with 29.4% of future cases expected to occur in this group. At this threshold, 5.3% of women were reclassified with the addition of density, leading to the identification of an additional 4.4% of future cases.

**Conclusion:**

Integrating density with questionnaire-based risk factors and PRS could potentially identify more women of European-ancestry with elevated risk of breast cancer in the United States and Sweden. Further investigations of the integrated model in non-European ancestry populations are needed prior to considering clinical applications.

## INTRODUCTION

Clinical application of risk-stratified breast cancer prevention strategies in the population requires the development and robust prospective validation of flexible and comprehensive models for absolute risk prediction to provide accurate individualized risk estimates, in particular for women at high-risk for whom such applications have the greatest potential impact ^1,2^. Several risk models have been developed incorporating different sets of risk factors and targeting different clinical scenarios ^3,4^; however, further work is needed to demonstrate whether improvements in risk stratification of current models may be achieved by incorporating additional risk factors, ultimately enhancing our ability to identify women at the extremes of risk distribution.

In our previous work, we built and validated a literature-based 5-year breast cancer prediction model incorporating reproductive, lifestyle and behavioral factors, family history and the recently developed PRS composed of 313 common variants ^5^ with the Individualized Coherent Absolute Risk Estimator (iCARE) software tool ^6^. This tool provides a flexible framework for absolute risk model development, aggregating information on risk factor associations, population-based age-specific disease incidence rates and competing mortality rates and the risk factor distributions from multiple data sources, and further implements standardized model validation methods. The model with questionnaire-based risk factors and PRS showed good calibration in multiple populations of European-ancestry women ^7^. Moreover, we also predicted that adding mammographic breast density to this model could further improve risk stratification ^8^.

Since the discovery by Wolfe in 1976 ^9^, density has been consistently shown to be a strong risk factor for breast cancer. The radio-opaque structures on a mammogram indicate stromal and epithelial tissue, while the radiolucent area indicates adipose tissue ^10^. Currently, the most widely used clinical system to classify density is the Breast Imaging-Reporting and Data System (BI-RADS), where density is visually assessed by a radiologist and categorized into 4 levels: almost entirely fatty, scattered areas of fibro-glandular density, heterogeneously dense, or extremely dense ^11^. Population-wide studies have demonstrated that approximately 50% of the US female population aged 40–74 have heterogeneously or extremely dense breasts, with extremely dense breasts conferring 2- to 4-fold higher relative risk compared to almost entirely fatty breasts ^12–15^.

Breast density has been incorporated into established risk models like the Gail model, the Breast Cancer Surveillance Consortium (BCSC) model, the Tyrer-Cuzick (IBIS) model, Rosner-Colditz model and the Breast and Ovarian Analysis of Disease Incidence and Carrier Estimation Algorithm (BOADICEA) ^16–23^. The BCSC, Tyrer-Cuzick and BOADICEA models also incorporate density into their clinical risk calculator tools 24–26. Robust evaluation of the added value of mammographic breast density to a model with comprehensive information on questionnaire-based risk factors and the most recent polygenic risk scores are needed to determine its effectiveness in improving risk stratification of models.

In our current work, we validate the fully integrated model with questionnaire-based risk factors, the 313-SNP PRS and density for calibration and discrimination in three prospective cohorts of European-ancestry women (two from the US and one from Sweden), totaling 1,468 cases and 19,104 controls. Our risk projection and reclassification calculations in the populations of European-ancestry women aged 50–70 years from US and Sweden show the improvements in risk stratification attainable by incorporating density to the current literature-based model with questionnaire-based risk factors and PRS.

## METHODS

### Study Populations

Model validation analyses were performed in three prospective cohort studies of European-ancestry women: US-based Nurses’ Health Study (NHS I and II) and Mayo Mammography Health Study (MMHS), and Sweden-based Karolinska Mammography Project for Risk Prediction of Breast Cancer (KARMA) study. In total, analyses were carried out in 1,468 cases and 19,104 controls and were done separately for women younger (NHS II, KARMA; 280 cases, 5,037 controls) and older than 50 years (NHS I, MMHS, KARMA; 1,188 cases, 14,067 controls). Women with a prior history of breast and other cancer, except for nonmelanoma skin cancer, were excluded from the study. Women consented for the use of their genetic material, mammogram with density and completed a risk factor questionnaire. For women with multiple mammograms or multiple questionnaires, the data closest to the DNA collection were used. In MMHS, density was obtained from routine clinical examination by attending radiologists. All four mammogram views (craniocaudal and mediolateral oblique for ipsilateral and contralateral sides) contribute to the assessment of density ^24^. In both NHS and KARMA, mammographic density was measured using semi-automated software, Cumulus ^25^ and STRATUS ^26^ respectively, and converted to a four-level variable to approximate density categories following BI-RADS ^27^. With Cumulus software, an area-based measure of mammographic density is estimated with user-defined thresholds to define dense tissue, and percent density (i.e., dense tissue area / total breast area) was categorized using the thresholds < 10%, 10–24% 25–49% and > 50% ^28,29^. With STRATUS, an area-based measure of mammographic density is estimated using a machine learning method. Thereafter, the percentage of MD is calculated as the ratio of dense tissue to the total area. This measure was then categorized to approximate BI-RADS categories using the thresholds < 2%, 2–8%, 8–49% and > 49% ^30^. Characteristics of the prospective cohort studies used for model validation and the distribution of risk factors is provided in the supplementary material (Tables S1 and S2, respectively).

Breast cancer outcome was ascertained through linkage to SEER registries, state tumor registries, and pathology databases. A woman was considered a case when she developed incident primary breast cancer, either *in situ* or invasive during the follow-up period. To reduce the possibility of screen-detected cancers, the first year of follow-up was omitted from validation analysis. Follow-up was defined as one year following study entry age up to the last record of cancer registry linkage or 5 years, whichever came first.

### Risk Model Development

We used iCARE ^6^ to build a model for 5-year absolute risk of developing breast cancer integrating questionnaire-based risk factors, PRS and density separately for women younger and older than 50 years. The questionnaire-based risk factors included were ages at menarche, first birth and menopause, parity, height, BMI, alcohol intake, family history (i.e., presence/absence of breast cancer in at least one first-degree relative), history of benign breast disease, oral contraceptive use, menopausal hormone therapy (MHT) use, and current MHT type. Our previous works ^7,8^ describe the integration of questionnaire-based risk factors and the 313-variant PRS ^5^ and here we further extend the model incorporating density. The relative risk estimate of the 4-level density variable was obtained through a literature search and was integrated assuming a multiplicative joint association with the other factors ^31^. For women younger than 50 the estimates were obtained from Tice et al. ^19^, and for women over 50 years from Barlow et al. ^32^.

### Risk factor distribution

An individual level reference dataset of risk factors, representative of the underlying country’s population, was used to estimate the joint distribution of risk factors and absolute risk projections in the respective population. For the US population, the majority of the risk factors were derived from the National Health and Nutrition Examination Survey (NHANES) from 2008, 2010, 2012 as previously described ^8^. The PRS was simulated assuming an independent relationship with the questionnaire-based risk factors, conditional on family history. Density was incorporated to the existing reference dataset using simulation based on a regression model with the questionnaire-based risk factors as predictors. The model parameters were estimated using data on 63,756 women from the Breast Cancer Surveillance Consortium (BCSC), as described in the supplementary materials. For the Swedish population, the reference dataset was generated using data from the controls in the prospective cohort KARMA. Subjects with no missing information for the risk factors were included in the reference dataset.

### Model Validation

The integrated model predicting 5-year absolute risk was prospectively evaluated for calibration and discriminatory accuracy. Calibration refers to the model’s ability to accurately predict the absolute risk of breast cancer. We evaluate calibration by estimating the ratio of expected-to-observed (E/O) number of cases, the calibration slope and intercept. We also show calibration plots after categorizing the subjects based on deciles of 5-year absolute risk.

The discriminatory accuracy of the model is assessed using the area under the receiver operating characteristic curve (AUC) based on the 5-year absolute risk. AUC is defined as the probability that for a case-control pair from the population, the risk for the case is higher than the risk for the control. An AUC of 50% corresponds to a model with no discriminatory power while 100% corresponds to perfect discrimination.

### Risk Reclassification

Improvements in risk stratification resulting from incorporation of density to a model with questionnaire-based risk factors and PRS were assessed among women of European-ancestry aged 50–70 years in the populations of US and Sweden by calculating the number of women and future cases identified to be at high-risk based on pre-specified 5-year absolute risk thresholds. We used two high-risk thresholds: 3%, which corresponds to the United States Preventive Services Task Force (USPSTF) recommendation for risk-reducing interventions ^33^, as well as 6%, which corresponds to the breast cancer risk of a BRCA mutation carrier and is used as a cutoff for very high risk by the WISDOM Trial ^34,35^. We also calculated the number of women and future cases re-classified at the high-risk thresholds to further quantify the improvements in risk stratification after incorporating density to a model with questionnaire-based risk factors and PRS.

All analyses were performed using R version 3.6.2 (www.r-project.org).

## RESULTS

### Model validation results

For women younger than 50 years, the integrated model with questionnaire-based risk factors, PRS and density was generally well calibrated in terms of relative and absolute risk across the risk categories in NHS II and KARMA. In the meta-analysis across studies, the area under the curves (AUC) with and without density were 67.0% (63.5%−70.6%) vs. 65.6% (61.9%−69.3%), respectively (Table S6A, Fig. 3).

**Figure 2:**
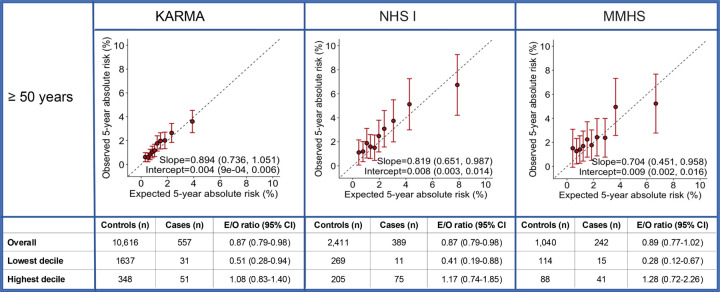
Calibration and discrimination of 5-year risk predictions of breast cancer for women aged 50 years and older Calibration and discrimination of 5-year risk predictions of breast cancer for women aged 50 years and older in the nested case-control sample of KARMA, NHS I and MMHS with risk categories based on deciles of predicted 5-year absolute risk. Validation results are shown for the extended iCARE model that incorporates questionnaire-based risk factors with a PRS based on 313 common germline variants and density. Estimates and 95% CI of the calibration slope and intercept are reported based on a linear regression of the decile-specific observed proportion of cases within 5 years and the average of the predicted 5-year absolute risk. AUC = area under the curve, *c*^2^ =chi-square goodness-of-fit test, CI = confidence interval, E/O = expected to observed number of cases, KARMA = Karolinska Mammography Project for Risk Prediction of Breast Cancer, MMHS = Mayo Mammography Health Study, NHS I = Nurses’ Health Study I, PRS = polygenic risk score, QRF = Questionnaire-based risk factors.

**Figure 3:**
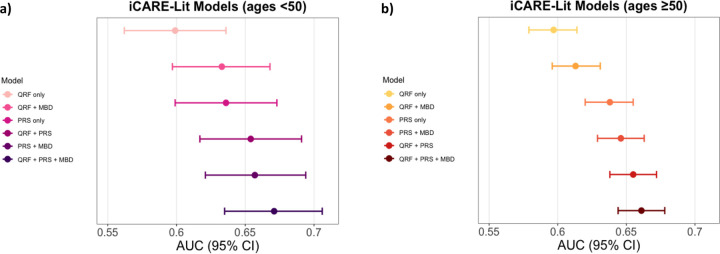
Risk discrimination measured by the model area under the curve (AUC) of the iCARE-Lit models Risk discrimination measured by the model area under the curve (AUC) of the iCARE-Lit models for a) women younger than 50 and b) 50 years or older based on a meta-analysis across studies for the risk-factor combinations: incorporates (i) questionnaire-based risk factors only, (ii) questionnaire-based risk factors with density, (iii) questionnaire-based risk factors with a PRS based on 313 common germline variants, (iv) the 313-variant PRS only, (v) the 313-variant PRS and density and (vi) the fully integrated model incorporating questionnaire-based risk factors, the 313-variant PRS and density. Colored dots were used to denote estimates and colored horizontal lines denote the 95% confidence intervals. iCARE-Lit = iCARE model based on literature review; MBD = mammographic breast density; PRS = polygenic risk score; QRF = questionnaire-based risk factors.

For women 50 years or older, the integrated model showed some underestimation of overall risk in NHS I and KARMA (NHS I: E/O = 0.87 (0.79–0.98) and KARMA: E/O = 0.87 (0.79–0.98), Table S5, Fig. 2). This was mainly noticeable in the lowest risk decile (KARMA: E/O = 0.51 (0.28–0.94), NHSI: E/O = 0.41 (0.19–0.88), MMHS: E/O = 0.28 (0.12–0.67), Fig. 2). For this age group, in the meta-analysis across studies, the AUCs with and without density were 66.1% (95% CI 64.4–67.8%) and 65.5% (95% CI: 63.8–67.2%) (Table S6B, Fig. 3).

### Risk stratification and reclassification

**Figure 4:**
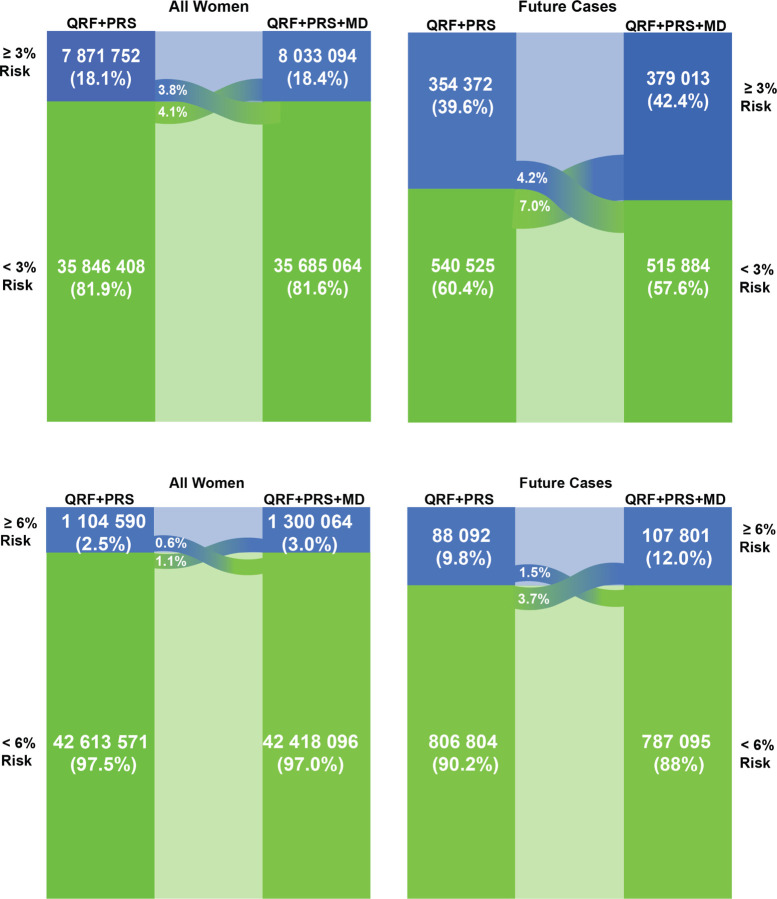
Reclassification of US women at two high-risk thresholds Women of European ancestry aged 50–70 years in the general population of the USA expected to be identified at high risk of breast cancer according to two risk thresholds, and the incident cases of breast cancer expected to occur in these groups within a 5-year interval, comparing the (i) questionnaire-based risk factor and PRS model with (ii) the fully integrated model with questionnaire-based risk factors, PRS and density. The expected number of women is calculated using 2020 (*N=* 43 718 160) population estimates from the US Census Bureau for the USA. The expected numbers of cases are estimated using the average predicted 5-year risk in the US population, calculated using the US breast-cancer-incidence rates and risk-factor distributions (Supplementary data). The 3% threshold is used by the US Preventive Services Task Force for recommending risk-reducing medications and 6% is used by the WISDOM trial as a cutoff for very high risk ^33,36^. AR = absolute risk, BMI = body mass index, MBD =mammographic breast density, PRS = polygenic risk score, QRF = Questionnaire-based risk factors, US = United States.

The extended model with questionnaire-based risk factors, the 313-variant PRS and density identified 18.4% of the of US non-Hispanic White population women 50–70 years old ≥ 3% predicted 5-year risk, the cut-off used for recommending risk-reducing medication in the US (Table S7, Fig. 4). This group includes 42.4% of predicted future cases (Table S7, Fig. 4). The addition of density led to the reclassification of 7.9% of US non-Hispanic White women aged 50–70 years, with 4.1% moving from below the ≥ 3% predicted 5-year risk threshold to above and 3.8% moving in the opposite direction. This resulted in the identification of 2.8% of additional future cases (Table S8, Fig. 4). At and above the 6% risk threshold, the fully integrated model identified 3.0% of the US non-Hispanic White population above 50–70 years. This group is expected to include 12.0% of future cases (Table S7, Fig. 4). Among these women, 1.7% were reclassified, with 1.1% moving from below the ≥ 6% predicted 5-year risk threshold to above and 0.6% moving in the opposite direction. This led to the identification of 2.2% additional future cases (Table S8, Fig. 4).

**Figure 5:**
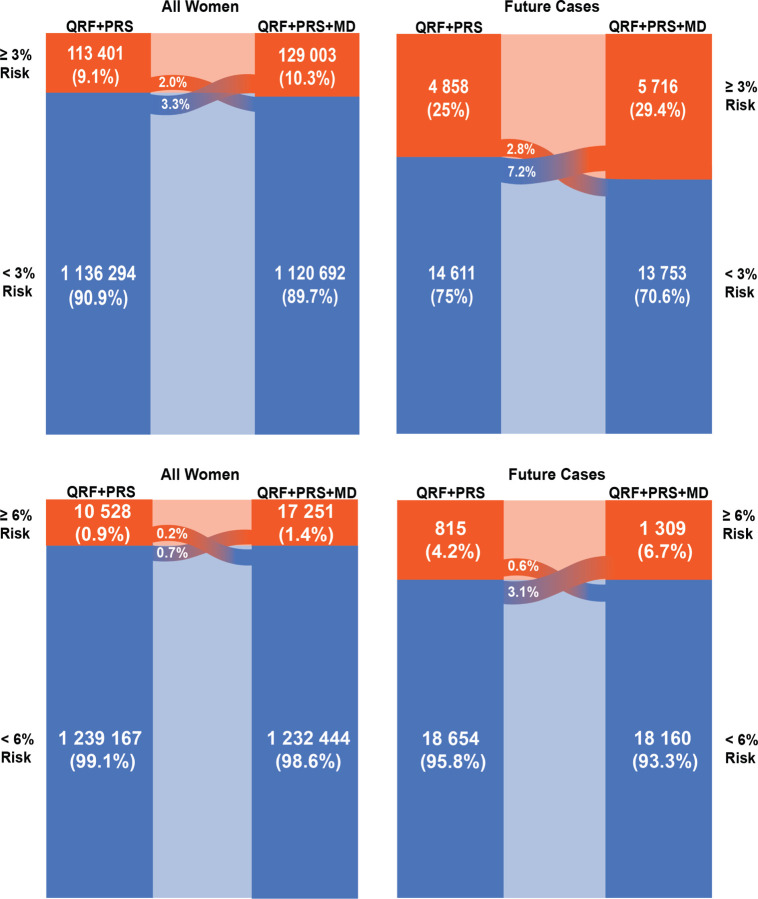
Reclassification of Swedish women at two high-risk thresholds Women of European ancestry aged 50–70 years in the general population of the USA expected to be identified at high risk of breast cancer according to two risk thresholds, and the incident cases of breast cancer expected to occur in these groups within a 5-year interval, comparing the (i) questionnaire-based risk factor and PRS model with (ii) the fully integrated model with questionnaire-based risk factors, PRS and density. The expected number of women is calculated using 2016 population estimates (N = 1 249 695) from Statistics Sweden for Sweden. The expected numbers of cases are estimated using the average predicted 5-year risk in the Swedish population, calculated using the SE breast-cancer-incidence rates and risk-factor distributions (Supplementary data). The 3% threshold is used by the US Preventive Services Task Force for recommending risk-reducing medications and 6% is used by the WISDOM trial as a cutoff for very high risk ^33,36^. AR = absolute risk, BMI = body mass index, MBD = mammographic breast density, PRS = polygenic risk score, QRF = Questionnaire-based risk factors, SE = Sweden.

In the Swedish population, the integrated model identified 10.3% of women aged 50–70 years ≥ 3% predicted 5-year risk with 29.4% of future cases expected to occur in this group (Table S7, Fig. 5). With the addition of density, 5.3% of women of European ancestry were reclassified, with 3.3% from below to above and 2.0% in the opposite direction. This identified an additional 4.4% of future cases (Table S8, Fig. 5). At and above the 6% risk threshold, the fully integrated model identified 1.4% of women of European ancestry aged 50–70 in the Swedish population. 6.7% of future cases are expected to occur in this group (Table S7, Fig. 5). With the addition of density, 0.9% of all women were reclassified above the ≥ 6% risk threshold, with 0.7% of women moving from below the threshold to above, and 0.2% moving in the opposite direction. This resulted in the identification of 2.5% additional future cases (Table S8, Fig. 5).

## DISCUSSION

We investigated the added value of incorporating breast density to the iCARE questionnaire-based risk factor and PRS model to predict 5-year absolute risk of breast cancer among women of European ancestry. Addition of density to the model resulted in a modest improvement in risk stratification and reclassification. For instance, incorporating density identified an additional ~ 3% of future cases at and above a 3% predicted 5-year risk threshold and an additional ~ 3–7% of future cases at and above a 6% predicted 5-year risk threshold in populations of European ancestry in the USA or Sweden. The model’s ability to stratify more women above and below clinically relevant risk thresholds would lead to more women being rightfully allocated to high and low-risk categories and therefore able to qualify for risk-reducing strategies.

The implementation of such risk-stratified screening and prevention strategies of breast cancer in the population is a key goal of risk prediction efforts. Currently, two non-inferiority trials, WISDOM and MyPeBS, are underway investigating the potential of risk-based personalized screening as a safe alternative to mammographic screening programs ^36^. As present screening programs are based solely on age as an entry criterion; comprehensive risk models can pave the way for individualized risk-based screening strategies. For countries where density is collected routinely in the clinical setting, this would be a relatively simple addition to risk models.

Our model incorporating density showed some signs of miscalibration at the extremes of the distribution. Since the relative risks were derived using a literature review, and is subject to assumptions about the underlying regression model. For women younger than 50 years, we obtained an age-adjusted relative risk for density from Tice et al. ^19^ which did not account for the correlation of density with BMI ^37^. Moreover, in both NHS and KARMA, mammographic density was measured using semi-automated software, CUMULUS and STRATUS respectively, and converted to a four-level variable to approximate density. There might be some misclassification due to the conversion from percent density to the four category visually assessed density. Conversely, in MMHS the density categorization might have contributed to misspecification as it was visually assessed by radiologists, and although broadly accepted, the BI-RADS reporting system is subject to substantial intra- and inter-observer variability between radiologists, with Kappa values ^38^ ranging between 0.4–0.7 ^40–43^. Several studies show a stronger relationship between breast cancer and percent density compared to BI-RADS density ^39–42^. Incorporating automated quantitative mammographic features of mammographic images into risk models could address these limitations. Interestingly, Kerlikowske et al. ^43^ found similar discriminatory accuracy between their model with automated or clinical density and Brentnall et al. ^44^ found that adding both a clinical and automated volumetric density measure improved risk stratification.

Moreover, we used data from prospective cohorts of women of European ancestry in the USA and Sweden that may not entirely representative of the general population. Although this could influence calibration of absolute risk, the relative risk calibration of the models is unlikely to be substantially affected. Analyses of risk distribution and re-calibration in the USA and Swedish populations used nationally representative data on incidence rates and risk factor distribution, and therefore are relevant to these target populations. Additional research is needed towards developing and validating risk prediction models for women of non-European ancestry for the risk-stratified prevention of breast cancer for those women.

This paper has a notable strength in the breadth of the validation, as it assesses the model’s performance across three different studies with participants coming from two populations of European-ancestry women in the US and Sweden. Other validation studies either included smaller number of participants or used combinations of a limited set of risk factors and polygenic risk scores (PRS) with fewer genetic variants (Table S9) ^16,20,45–47^. Recently, the extended version of the BOADICEA model incorporating questionnaire-based risk factors, the 313-SNP PRS, density and rare moderate- and high-risk variants has also been externally validated in KARMA, similarly showing the importance of reclassification and risk stratification ^45^. Other established models have also shown improvement of model discrimination after the incorporation of density ^44,46,48–51^, however most have yet to be externally validated in fully independent cohorts. The independent external validation of risk models is critical before clinical applications; however, a potential barrier is availability of large prospective cohorts with comprehensive genetic, mammography and risk factor information. The Tyrer-Cuzick (v 7.02) model and the BCSC model (v2) with mammographic density have been validated in large independent cohorts, however both these versions do not include genetic information ^16,22^. In contrast to the three aforementioned validation studies, other established models like the Gail model have only been internally validated after the addition of breast density information ^21,48^. Moreover, there is an increasing interest in using fully automated density measures using deep learning algorithms ^52,53^. Using artificial intelligence on mammograms to aid risk prediction has the benefit of moving beyond density by also characterizing (micro)calcifications, focal masses, left-right asymmetry and a landscape of radiomic features ^54–56^. However, many of these algorithms have yet to be independently validated in epidemiological and clinical studies.

To summarize, the incorporation of density to questionnaire-based risk factors and PRS results in modest improvements in identification of European-ancestry women at elevated breast cancer risk. Additional prospective validation in diverse populations, in particular of non-European ancestry women, are needed to ensure equitable clinical application.

## Supplementary Material

Supplement 1

## Figures and Tables

**Figure 1:**
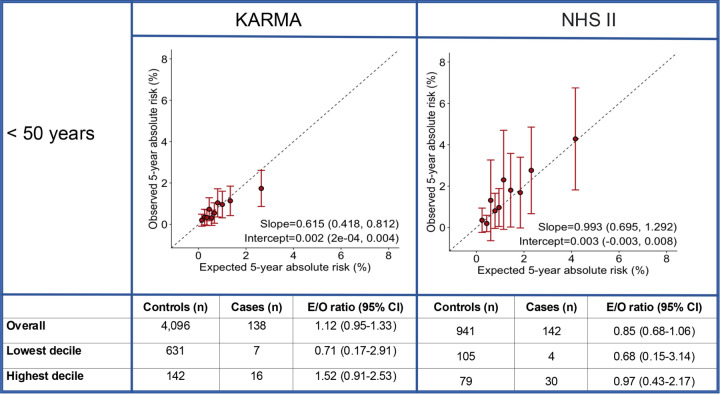
Calibration and discrimination of 5-year risk predictions of breast cancer for women aged younger than 50 years Calibration and discrimination of 5-year risk predictions of breast cancer for women aged younger than 50 years in the nested case-control sample of NHS II and KARMA with risk categories based on deciles of predicted 5-year absolute risk. Validation results are shown for the extended iCARE model that incorporates questionnaire-based risk factors with a PRS based on 313 common germline variants and density. Estimates and 95% CI of the calibration slope and intercept are reported based on a linear regression of the decile-specific observed proportion of cases within 5 years and the average of the predicted 5-year absolute risk. AUC = area under the curve, *c*^2^ =chi-square goodness-of-fit test, CI = confidence interval, E/O = expected to observed number of cases, KARMA = Karolinska Mammography Project for Risk Prediction of Breast Cancer, NHS II = Nurses’ Health Study II, PRS = polygenic risk score, QRF = Questionnaire-based risk factors.

## Data Availability

The data sets used in the current analysis will not be made publicly available due to restraints imposed by the ethics committees of individual studies; requests for individual-level data for all the participants in the full cohort of any study can be made to the individual studies.
