# Supplementary material for: Investigating the added value of incorporatingmammographic density to an integrated breastcancer risk model with questionnaire-based riskfactors and polygenic risk score
Source: Res Sq. 2024 Dec 19:rs.3.rs-5445786. Preprint. [Version 1] doi: 10.21203/rs.3.rs-5445786/v1 (PMC11702789; doi:10.21203/rs.3.rs-5445786/v1)
Supplement: Supplement 1 [file nihpprs5445786v1-supplement-1.pdf]

Supplementary Files

This is a list of supplementary files associated with this preprint. Click to download.

- 20240418supplementarymaterial.docx
